# Comparison of Perimetric Outcomes from Melbourne Rapid Fields Tablet Perimeter Software and Humphrey Field Analyzer in Glaucoma Patients

**DOI:** 10.1155/2020/8384509

**Published:** 2020-08-22

**Authors:** Harsh Kumar, Mithun Thulasidas

**Affiliations:** Glaucoma Services, Centre for Sight, B-5/24, Safdarjung Enclave, New Delhi 110029, India

## Abstract

**Purpose:**

To compare visual field results obtained using Melbourne Rapid Fields (MRF) iPad-based perimeter software and Humphrey Field Analyzer (HFA) 24-2 Swedish Interactive Threshold Algorithm (SITA) standard program in glaucoma patients.

**Design:**

A cross-sectional observational study.

**Methods:**

In this single-centre study involving patients diagnosed with glaucoma, the perimetric outcomes of MRF were compared against those returned from the HFA 24-2 SITA standard. Outcomes included mean deviation (MD), pattern standard deviation (PSD), visual field index (VFI)/visual capacity (VC), foveal threshold, test time, number of points depressed at *P* < 5% on PSD probability plot, and glaucoma hemifield test/color coded indicator.

**Results:**

The study included 28 eyes of 28 glaucoma patients. Mean (standard deviation) test times were 342.07 (56.70) seconds for MRF and 375.11 (88.95) for HFA 24-2 SITA standard (*P*=0.046). Mean MD was significantly lower for MRF (Δ = 3.09, *P* < 0.001), and mean PSD was significantly higher for MRF (Δ = 1.40, *P*=0.005) compared with HFA. The mean foveal threshold for the MRF was significantly lower than the mean HFA foveal threshold ((Δ = 9.25, *P* < 0.001). The number of points depressed at *P* < 5% on the PSD probability plot was significantly less for MRF (*P* < 0.001). Other perimetric outcomes showed no significant differences between both. Bland–Altman plots showed that considerable variability existed between the programs.

**Conclusion:**

MRF is a good cost-effective, time-saving, user-friendly tool for monitoring visual fields in settings where access to traditional perimetry is limited. The lack of Internet strength in rural areas and questionable detection of early cases may be two points in MRF fields requiring an upgrade.

## 1. Introduction

Glaucoma is a leading cause of irreversible blindness worldwide characterized by permanent loss of retinal ganglion cells and visual field damage [1]. Visual field testing is the only direct method by which the functional defects in glaucoma can be measured though it gives inconsistent responses especially in perimetrically inexperienced patients and has a controversial impact on intraocular pressure levels [2–7]. Standard automated perimetry is currently the most common and preferred method to evaluate functional defects in clinical glaucoma practice despite the well-known drawback of subjective testing. Another novel technique of estimating visual sensitivities at retinal points is microperimetry, which is also known as fundus-driven perimetry [8, 9]. Microperimetry has been used to assess visual function in a few clinical studies of glaucoma, although it is limited to the evaluation of macular rather than peripheral retinal sensitivity [10–12].

The Humphrey Field Analyzer (HFA) 24-2 Swedish Interactive Threshold Algorithm (SITA) standard program is widely accepted and can provide reasonably accurate, reliable, and reproducible visual field outcomes in patients with glaucoma [13, 14]. However, it is not easily portable, and the cost of the device restricts its availability to patients from underprivileged places and those in rural and remote areas. It also requires the presence of trained personnel to set up and run the testing program. Touch screens and tablet devices such as iPad have provided new opportunities for alternative forms of testing [15, 16]. The development of a tablet-based perimeter follows from an early suprathreshold test developed on iPad generation 1 (Visual Field Easy app), which was observed to be useful in visual field screening in Nepal [17]. The Melbourne Rapid Fields (MRF; FDA cleared class 1 device) is an iPad-based perimeter application (iPad 3 or later) that allows in-office or remote visual field testing due to its low cost and easy portability. MRF has been shown to produce comparable results to the HFA and has good intrasession test-retest repeatability [18–21]. It has been shown to be robust to variations in ambient light, blur, and viewing distance [18].

The purpose of the present study was to compare visual field results obtained using MRF iPad-based perimeter software and HFA 24-2 SITA standard program in patients with glaucoma in a clinical setting in North India. The present study is also useful to understand the potential of an alternative method to “bowl perimetry” due to concerns regarding the decontamination of “bowl perimetry” in the current coronavirus disease 2019 (COVID-19) pandemic period [22]. The highlights of this study include its exclusive study population of Asian origin and identification of new aspects of MRF software that need further development.

## 2. Materials and Methods

A cross-sectional observational study was conducted at the Centre for Sight Eye Hospital, New Delhi, India, to compare MRF iPad-based perimeter software and HFA 24-2 SITA standard program in patients with glaucoma. Informed consent was obtained from each patient according to the tenets of the Declaration of Helsinki.

Inclusion criteria included patients between 20 and 70 years of age having a diagnosis of glaucoma, previous experience with threshold perimetry on HFA, visual acuity ≥6/18, the spherical equivalent refractive error between -4 dioptres (D) and +4D, and astigmatism ≤2.5D. Glaucoma diagnosis was based on the presence of reproducible visual field defects on the HFA 24-2 SITA standard program with corresponding structural defects in the optic disc on clinical examination. Patients who were unable to do the perimetry test due to any systemic illness, patients with ocular disease other than glaucoma, or patients with the neurological disease were excluded.

### 2.1. MRF Strategy

**Figure 1:**
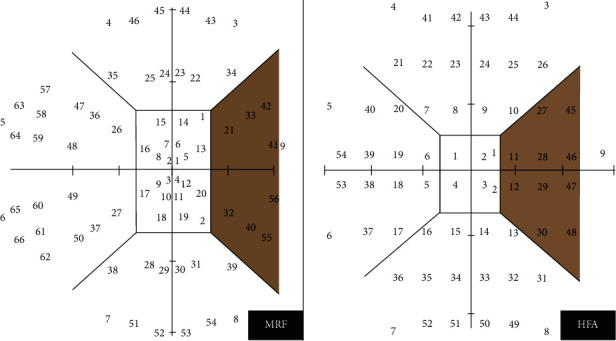
Nine zones with 66 test spots for the Melbourne Rapid Fields (MRF) full test and 54 test spots for the Humphrey Field Analyzer (HFA) 24-2 grid.

**Figure 2:**
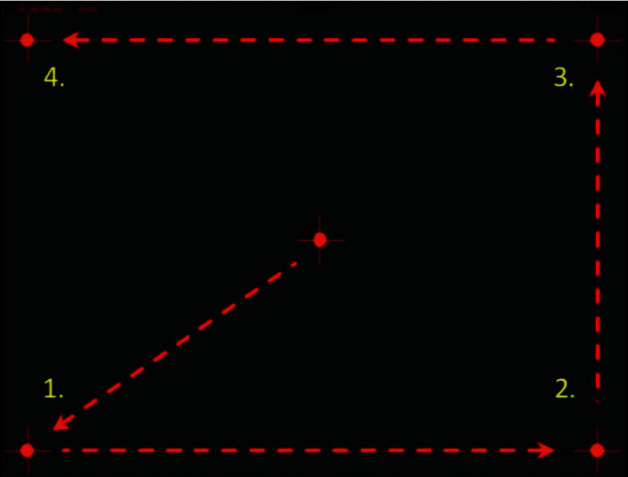
Fixation changes during Melbourne Rapid Fields (MRF) testing (for 9.7″, 10.5″, and 11″ iPads).

The iPad luminance output was calibrated automatically by the MRF software before starting the test and was found to deliver approximately 31 decibels (dB) of operating range on a 5 cd·m^−2^ background, giving a dynamic range close to that of HFA visual fields (35 dB) [18]. The MRF perimeter software used a modified 24-2 grid or a full test centred at fixation, which could test 30° × 20° of the visual field, equivalent to the HFA 24-2 program. In the radial pattern full test, 66 locations were used (Figure 1 compared with HFA 24-2 grid). The software controls the difference in number and location as per the selected test. As the screen size was small, subtending around 15 × 12°, the patient had to fixate in the four corners of the screen at different times to gain eccentricities out to 30° (Figure 2). The spot size increased with eccentricity to account for the tangent effect of the flat screen and to produce a fixed threshold across the central field (instead of a hill of vision). Targets were presented for 300 milliseconds (ms). The thresholding strategy started with a 17 dB stimulus and used a three-presentation binary Bayesian protocol to yield eight steps (0, 3, 6, 12, 17, 22, 26, and 30 dB) across the 30 dB range (Zippy Estimation by Sequential Testing, ZEST). The protocol tested additional intermediate points adjacent to a detected area of scotoma [20].

Fixation monitoring was implemented with a blind spot monitor using a 19 dB stimulus approximately 40% larger in area than Goldmann size II spot. Testing was performed by locating the blind spot at the beginning of each test, and a stimulus was presented (8–10 times) in that location throughout the test using central fixation. False-positive and false-negative checks were presented throughout the test. False-positive checks were conducted by interspacing periods (1000–1400 ms) throughout the test, during which no stimulus was presented on the screen. A false-positive was recorded if the patient gave a response during that period [19].

Voice prompts for the test procedure were provided in multiple languages by the iPad software to guide the patient during the test. In the present study, we have used a 9.7-inch 12.1.1 iOS version iPad and chosen the English language to provide voice prompt for all patients. The response of the patient to the presentation of a stimulus could be recorded by touching the screen or the spacebar on a Bluetooth keyboard connected to the iPad device. Touching the spacebar on the Bluetooth keyboard method was used in the present study to keep the screen clear without any fingerprints. Also, better tactile feedback was provided to the patient on making the response [19].

**Figure 3:**
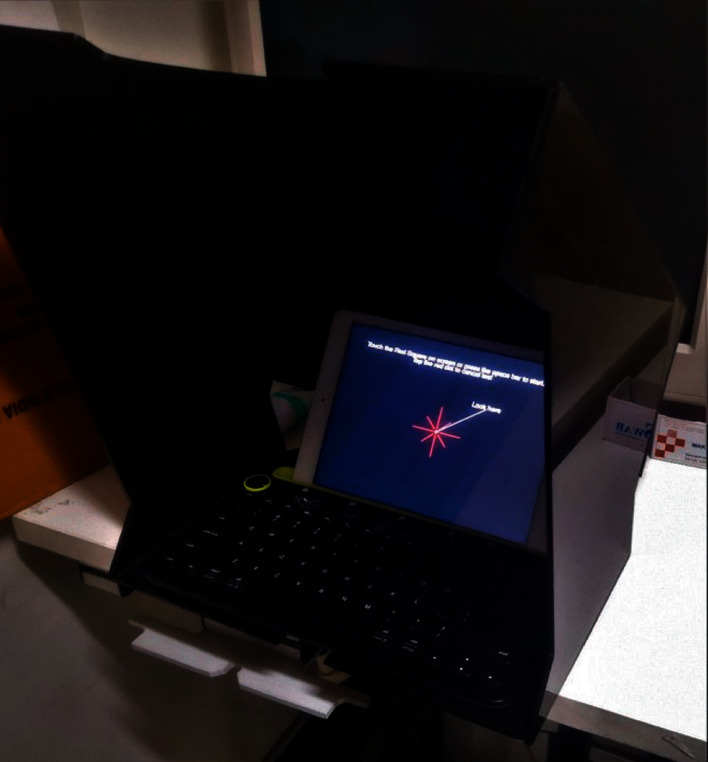
The iPad-keyboard-viewing-shield apparatus with a headrest maintaining 33 cm viewing distance for Melbourne Rapid Fields (MRF) testing.

**Figure 4:**
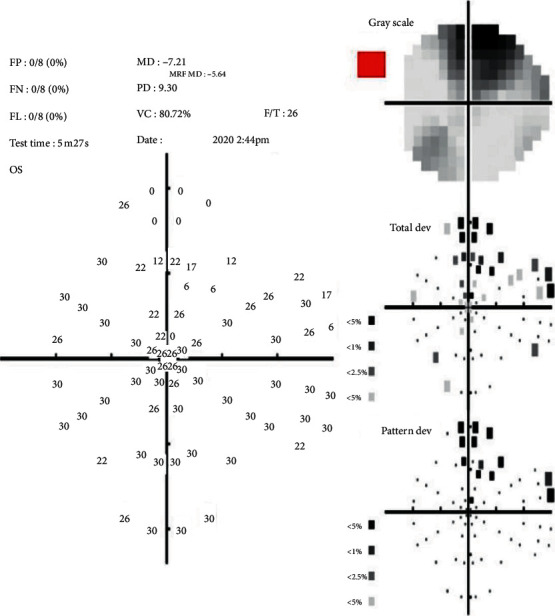
Visual field output for Melbourne Rapid Fields (MRF) full test.

One eye of each patient was randomly included in the study. All patients were new to MRF iPad-based perimeter software. Testing was performed with natural pupils. The patient was asked to wear his/her habitual reading glasses (monofocal, bifocal, or multifocal) as needed for normal near viewing. Before starting the testing, the clinician explained the test to the patient and what they were expected to do, including not to move their head position during the test. The fellow eye was occluded, and the patient was seated comfortably at a table with the iPad tablet placed on a typing stand that accompanied the keyboard and the viewing-shield with a headrest maintaining 33 cm viewing distance (Figure 3). Testing was conducted in a dim and evenly lit room with no direct reflections of doorways or windows on the screen. A short demo test lasting for approximately 1 minute was conducted to ensure that each patient understood the test and had become familiar with the voice prompts and response method used by the MRF. The clinician administering the test ensured that the 33 cm viewing distance was maintained throughout the test. If a change in viewing distance was noted, the test was paused, and the patients were repositioned. Patients were prompted to maintain fixation on the small red cross, which changed location with time. The iPad screen intensity was automatically set to 100% for each test by the software, and the iPad was turned on for at least 10 minutes before testing to ensure the stability of the luminous output. Color-coded indicator classified the fields into “Green” within normal limits (95% of normals), “Amber” borderline (<5% of normals), and “Red” outside normal limits (<1% of normals).  Figure 4 shows the visual field output for the MRF full test.

On the same day, each patient underwent HFA 24-2 SITA standard perimetric testing on a single Humphrey® field analyzer 3 perimeter (Carl Zeiss Meditec AG, Jena, Germany) with a time interval of at least half an hour after the MRF iPad perimetry test. Study eyes did not undergo tonometry before field testing. Only reliable HFA fields were considered for analysis (<20% fixation loss, <20% false-positive, and <20% false-negative).

Mean deviation (MD), pattern standard deviation (PSD), visual field index (VFI) for HFA/visual capacity (VC) for MRF, foveal threshold, test time, number of points depressed at *P* < 5% on PSD probability plot, and glaucoma hemifield test for HFA/colour coded indicator for MRF were assessed for each test.

### 2.2. Statistical Analysis

Statistical analysis was performed on the Statistical Package for Social Sciences (SPSS) version 24.0 for windows. Continuous variables were presented as mean ± standard deviation (SD). Categorical variables were presented as frequency and percentage. The Kolmogorov-Smirnov test tested the normality of the data. Quantitative variables between MRF and HFA were compared using paired *t*-test and Wilcoxon signed rank test. Qualitative variables were compared using the Chi-square test. The agreement between the programs for various parameters was done with Intraclass Correlation (ICC) and Bland–Altman plots. *P* value less than 0.05 is considered as significant at a 95% confidence level.

## 3. Results

28 eyes of 28 patients were included in this study. The mean age was 52.28 ± 14.76 years, ranging from 21 to 72 years. 17 (60.7%) patients were males.  Table 1 shows the comparison of visual field test parameters for MRF and HFA 24-2 SITA standard program. The mean test duration for the MRF was 342.07 ± 56.70 seconds, which was shorter than the average HFA 24-2 SITA standard test time for these same patients of 375.11 ± 88.95 seconds (*P*=0.046). The MRF test duration included the time required for the iPad to play voice prompts and for the patient to move their fixation when tested in the periphery. Compared to HFA, the mean MD was significantly lower for MRF with a mean difference of 3.09 ± 3.28 dB (*P* < 0.001), and mean PSD was significantly higher for MRF with a mean difference of 1.40 ± 2.15 dB (*P*=0.005). The mean foveal threshold for the MRF was 25.18 ± 7.01 dB, which was significantly lower than the mean HFA foveal threshold of 34.43 ± 3.17 dB (*P* < 0.001). The mean VC of MRF was compared with VFI of HFA, and no significant difference was found. The number of points depressed at *P* < 5% on the PSD probability plot was significantly less for MRF with a mean difference of −6.00(*P* < 0.001).  Table 2 shows the glaucoma hemifield test/colour coded indicator results obtained with HFA 24-2 SITA standard and MRF, where no significant difference was found. Reliability indices also showed no significant differences between MRF and HFA though all patients were new to MRF. In 4 (14.3%) eyes, MRF could not detect the blind spot.

**Figure 5:**
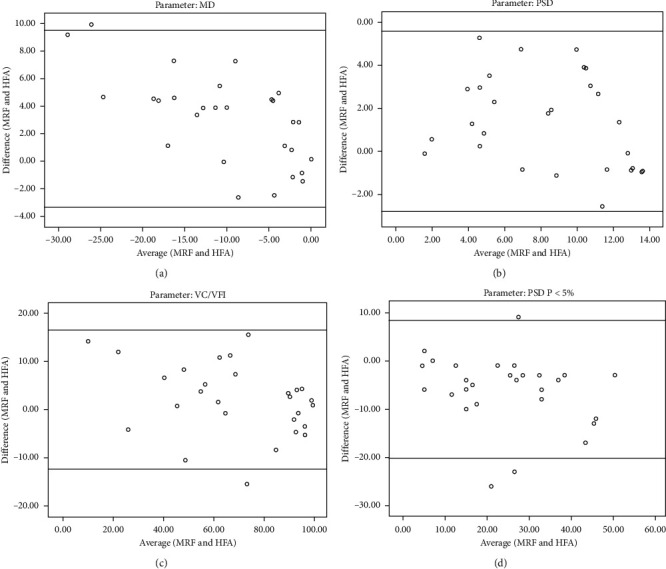
Bland–Altman plots showing agreement in (a) mean deviation (MD), (b) pattern standard deviation (PSD), (c) visual field index (VFI)/visual capacity (VC), and (d) the number of depressed points with *P* < 5% on PSD probability plot between Humphrey Field Analyzer (HFA) 24-2 Swedish Interactive Threshold Algorithm (SITA) Standard and Melbourne Rapid Fields (MRF).

A strong correlation was observed for MD (ICC = 0.928), PSD (ICC = 0.889), VFI/VC (ICC = 0.978), and depressed points with *P* < 5% (ICC = 0.882) on PSD probability plot between MRF and HFA.  Figure 5 provides the Bland–Altman plots showing agreement in MD, PSD, VFI/VC, and the number of depressed points with *P* < 5% on PSD probability plot between HFA 24-2 SITA standard and MRF. These plots demonstrated clearly that considerable variability could exist between the programs and can be appreciated visually by observing the graphs and mathematically by the 95% limits of agreement.

## 4. Discussion

Portable tablet devices such as iPad are useful in testing visual acuity, contrast sensitivity, and estimating retinal sensitivity in foveal locations [23–25]. The extension of a portable tablet device to visual field testing has the potential to allow detection and management of glaucoma in remote communities where access to traditional perimetric machines is limited. It will allow future use of such devices in terms of home monitoring, reducing resource burden on clinics, and allowing frequent field testing to provide earlier detection of glaucoma [26]. Only a few studies have compared the perimetric results of MRF iPad-based application with HFA, mostly including the Caucasian population [19–21]. We present a cross-sectional observational clinical study comparing perimetric parameters from MRF and HFA exclusively in the Indian population.

In the present study, the test time of the MRF was approximately 0.5 minutes/eye shorter than HFA 24-2 SITA standard when conducted on the 9.7-inch iPad, which is consistent with other studies [19–21]. This may be due to fewer fixational movements needed with MRF for performing the test. Lesser test times are significant where there are limited time and resources available for a large group of patients, facilitating home monitoring by patients. It also helps old and debilitated patients as also those with issues of pressing a button due to arthritic problems.

The MRF showed significantly lower MD and higher PSD compared to HFA, which is probably due to the difference in the number, size, location, and duration of stimuli used in MRF [27]. Also, the Bayesian method used in MRF differs from the point-wise approach of HFA. In the point-wise approach, the user must estimate sensitivity at each point in the visual field because they differ in the retinal location. In MRF, the threshold is constant at all 66 locations (30 dB). The difference in the baseline age-corrected normal threshold values may be another reason for the lower MD observed with MRF [28]. The mean foveal threshold was significantly lower for MRF with a mean difference of −9.25 dB, which may also be probably due to the difference in the number of stimulus presentations. This difference in foveal threshold may affect the individual threshold test points too, and this parameter was compared only in our study. The visual field index of HFA is termed as “visual capacity” in MRF, and no significant difference was found in the values between the two programs, similar to the observation by Schulz et al. [20].

In our study, MRF showed a significantly lesser number of points depressed at *P* < 5% on PSD probability plot than HFA, pointing towards the possibility of underestimating glaucomatous defects and missing early cases of glaucoma. This is in comparison with the finding by Schulz et al., where they stated that the MRF test missed some very early glaucoma cases [20]. Kong et al. in their study suggested that the MRF performed well in quantifying mild field defects, even though the agreement between MRF and HFA for mild defects was found to be less robust [19]. Prea et al. stated that the MRF test might be limited in detecting early progression, especially in regions with some loss of threshold [21].

The MRF provided standard reliability indices of false-positive, false-negative, and fixation losses. In the present study, both false-positive and false-negative rates were acceptable, with only 6 (21.4%) patients having false-positives and false-negatives >12%. In contrast, the rate of fixation losses in our study tended to be on the higher side, with 13 (46.4%) patients having fixation loss >12%. Fixation loss monitoring depends on accurate detection of a patient's blind spot, and MRF was unable to detect the blind spot in approximately 14% of the patients in this study. This is in comparison with the data reported by Schulz et al., where MRF could not detect blind spots in 18% of the glaucoma cohort [20].

An excellent agreement was observed between MRF and HFA for MD (ICC = 0.928), PSD (ICC = 0.889), and VFI/VC (ICC = 0.978), consistent with the findings of other studies [19–21]. However, assessing the degree of agreement using correlation coefficients may be sometimes misleading and inappropriate. Hence, we have also used Bland–Altman analyses in this study, which show the magnitude of the difference the clinician can expect between the algorithms in most (95%) instances, excluding the most atypical outliers [29]. The plots confirmed lower MD, higher PSD, and lesser test points depressed at *P* < 5% with MRF compared to HFA. Kong et al. also confirmed less negative MD returned by MRF using Bland–Altman analysis [19]. Schulz et al. and Prea et al., on the other hand, found little bias and an acceptable degree of scatter variability between MRF and HFA [20, 21]. This difference in the findings may be due to the variation in the study group or participants. In our study, we have included only glaucoma patients with reproducible field defects, whereas Schultz et al. had included preperimetric subjects, and Prea et al. had included participants with ocular hypertension in their studies [20, 21]. Though there is a strong agreement between HFA and MRF for VFI/VC, the programs could not be used interchangeably for the same patient on different test sessions unless some correction factor is added to account for the differences. Also, the field defect should be confirmed by repeat testing using the same program for an individual patient.

Most of the patients found the MRF test easy to perform. The lack of learning curve may indicate the experience patients already had with HFA perimetry. Few patients feel extremely claustrophobic in the bowl perimeter, and an open space test on the iPad is much more convenient for them. However, few patients with a history of cervical pain had difficulty bending the neck while performing MRF. In this study, no significant head movement was observed as the forehead support (headrest) might have stabilized the patient during the test. Vingrys et al. reported that significant forward and backward movement did not significantly influence threshold scores, but they also used a headrest for testing [18]. In the present study, the test was conducted in a dim and evenly lit room to minimize reflected light on the screen, as proposed by Vingyrs et al. [18]. We believe that the presence of viewing-shield may allow the test to be performed with room lights on, as it minimizes the effect of external light and veiling glare that can reduce spot visibility.

MRF is also cost-effective compared to HFA, as all we need to do is to buy an iPad (9.7″/10.5″/11″/12.9″) and install the software to perform the test. The iPad cost approximately ranges from $200 to $1150, depending upon the model. The software license fee is about $265, which allows the buyer to use it for a lifetime. The test package comes in multiple of 100 tests for around $330 per package, with each individual test consisting of one full visual field test 24-2, one macula test 10-2, and acuity test of high contrast and low luminance for both eyes. Other accessories include a keyboard that costs around $25 and a viewing-shield apparatus provided free of cost.

Some features of the MRF application require further attention and development. The iPad uses a lower background luminance than HFA to increase the dynamic range. One of the problems with iPad testing that will remain an issue in the community is the lack of standardized background illumination. However, the contrast ratio between background and stimulus luminance is more critical and has shown to be standard among tablets as well as different devices of the same model. The development of an effective tracking system for monitoring head and eye positions in real-time using the iPad camera may allow fixation monitoring during peripheral field testing as well as for the central test. MRF now uses voice prompts to remind patients to maintain fixation, but this is not a practical solution. Though voice prompts are available in multiple languages in the current version of MRF, the inclusion of more regional languages may be an advantage for screening in rural community settings. The provision of an Internet-free feature will be better before implementing MRF for remote community testing. The unavailability of the Internet in the peripheral areas may be a challenge in southeast Asian nations, and the change in the set-up with no Internet requirements would be appropriate. Also, the difficulty in posture faced by few patients can be improved by attaching a chinrest to the iPad-keyboard-viewing-shield apparatus in addition to the headrest.

Given the COVID-19 pandemic situation, the MRF software has recently launched a web version, where patients can perform visual field testing in the home environment on a laptop or television screen on their own. However, a lack of supervision by a clinician to ensure strict control of viewing distance and viewing environment should be considered while performing the test out of the clinical setting. The performance of patients who do not have experience in visual field testing will also need to be evaluated in terms of learning how to use a tablet in both supervised (clinic) and unsupervised (home) environments. To date, only a simulation study has been published on what might be expected from home-based testing [30].

Any new development or technique in the field must be compared with the gold standard system. This is the reason why we have compared MRF software with HFA in the present study. At no point in time, it should be deemed to replace MRF for HFA, the current gold standard. MRF can be used extensively for screening so that in a community, at least the moderate-advanced glaucoma cases can be detected and referred for complete management. Also, in the present COVID-19 era, where “bowl perimetry” decontamination is a concern, MRF has a potential advantage.

The strengths of our study include its exclusive study population of Asian origin, comparison of more perimetric parameters, and the identification of new aspects or limitations of MRF which need further development. Limitations of our study include its small sample size and lack of repeatability check on a long-term follow-up. Further prospective studies with a larger sample size to look for repeatability, test-retest variability, and home-based testing may provide more significant results.

In conclusion, despite using a completely different test paradigm, MRF perimetry provided reasonable perimetric outcomes compared to HFA, though few features needed further development. MRF is a good cost-effective, time-saving, user-friendly tool for monitoring visual fields in community settings where conventional perimetry is not readily accessible. Thus, even though some early glaucoma cases may be overlooked, detecting even moderate and advanced glaucoma in the remote community at a fraction of the cost justifies its further refinement and use. Lastly, MRF and HFA cannot be used interchangeably on different test sessions for the same patient.

## Figures and Tables

**Table 1:** Comparison of various test parameters for Melbourne Rapid Fields (MRF) and Humphrey Field Analyzer (HFA) 24-2 Swedish Interactive Threshold Algorithm (SITA) standard program.

Test parameter	MRF (mean ± SD)	HFA 24-2 SITA standard (mean ± SD)	*P*
MD (dB) ^*∗*^	−8.61 ± 7.21	−11.71 ± 9.36	<0.001^‡^
PSD (dB) ^*∗*^	9.09 ± 3.57	7.68 ± 4.19	0.005^‡^
VC (for MRF)/VFI (for HFA) (%) ^*∗*^	70.63 ± 24.56	68.43 ± 27.08	0.096
Foveal threshold (dB)^†^	25.18 ± 7.01	34.43 ± 3.17	<0.001^‡^
Test time (seconds)^†^	342.07 ± 56.70	375.11 ± 88.95	0.046^§^
Depressed points with *P* < 5% on PSD probability plot (n) ^*∗*^	21.61 ± 12.86	27.61 ± 14.51	<0.001^‡^

MD = mean deviation, PSD = pattern standard deviation, VC = visual capacity, VFI = visual field index, MRF = Melbourne rapid fields, HFA = Humphrey field analyzer, SITA = Swedish interactive thresholding algorithm, SD = standard deviation.  ^*∗*^Wilcoxon signed rank test analysis. ^†^Paired *t*-test analysis. ^‡^Highly significant with level of significance: *P* < 0.01. ^§^Significant with level of significance: *P* < 0.05 at 95.0% confidence limit.

**Table 2:** Glaucoma hemifield test/colour coded indicator results obtained with Humphrey Field Analyzer (HFA) 24-2 Swedish Interactive Threshold Algorithm (SITA) standard and Melbourne Rapid Fields (MRF).

Colour coded indicator/glaucoma hemifield test classification	MRF (n)	HFA 24-2 SITA standard (n)
Red/outside normal limits	26	28
Amber/borderline	1	0
Green/within normal limits	1	0

MRF = Melbourne rapid fields, HFA = Humphrey field analyzer, SITA = Swedish interactive thresholding algorithm.

## Data Availability

The data used to support the findings of this study are available from the corresponding author upon request.
